# Impact of dietary Mannan-oligosaccharide and β-Glucan supplementation on growth, histopathology, E-coli colonization and hepatic transcripts of *TNF-α* and *NF- ϰB* of broiler challenged with *E. coli* O_78_

**DOI:** 10.1186/s12917-020-02423-2

**Published:** 2020-06-19

**Authors:** Sabreen Ezzat Fadl, Ghada Ahmed El-Gammal, Osama Atia Sakr, Aly A. B. S. Salah, Ayman Ali Atia, Abdelbary Mohammed Prince, Abdelhaleem Mohamed Hegazy

**Affiliations:** 1 Biochemistry Department, Faculty of Veterinary Medicine – Matrouh University, Matrouh, Egypt; 2 Microbiology Unit, Animal Health Res. Inst. (ARC), Kafrelsheikh, Egypt; 3 Biochemistry, Nutritional Deficiency Diseases and Toxicology Unit, Animal Health Res. Inst. (ARC), kafrelsheikh, Egypt; 4 Pharmacology Unit, Animal Health Res. Inst. (ARC), Kafrelsheikh, Egypt; 5 Pathology Unit, Animal Health Res. Inst. (ARC), Kafrelsheikh, Egypt; 6 Department of Molecular Biology, Faculty of Vet. Med., Cairo University, Cairo, Egypt; 7 Poultry diseases Unit, Animal Health Res. Inst. (ARC), Kafrelsheikh, Egypt

**Keywords:** Antioxidant, Gene expression, Growth performance, Histopathology, Immunostimulant, Agrimos

## Abstract

**Background:**

Using probiotics have become popular. They are considered an alternative to Antibiotic Growth Promoters (AGP). Probiotics are supplemented into animal feed for improving growth performance along with preventing and controlling enteric pathogens. The aim of this work was to study the impact of dietary supplementation of Mannan-oligosaccharide and β-Glucan (Agrimos®) on broiler challenged with *Escherichia coli* O_78_ (*E. coli* O_78_ - marked with an antibiotic (320 μg ciprofloxacin/ml broth) on growth performance, serum biochemistry, immune organs-histopathology, *E-coli* colonization, and hepatic transcripts of Tumor necrosis factor-alpha (TNF-α) and Nuclear factor-kappa B (NF-ϰB). A total of 125 one-day-old chicks were used for conducting the experiment. Five one-day-old chicks were slaughtered for measuring the initial weight of the lymphoid tissue. The remaining chicks (120) were allotted into four groups according to Mannan-oligosaccharide and β-Glucan supplementation, and *E. coli* infection. The data were analyzed using SPSS version 16.

**Results:**

Results indicated significant alteration of growth performance, serum biochemistry, and selected liver gene expression with pathological lesions, especially in the lymphoid organs due to *E. coli* infection. These alterations were mitigated by Mannan-oligosaccharide and β-Glucan supplementation.

**Conclusion:**

It could be concluded, Mannan-oligosaccharide and β-Glucan supplementation in broiler’s diet improved the immune response of broilers and mitigated pathological lesion resulted from *E. coli* infection.

## Background

The increasing demand for animal protein worldwide led to a gap, thus searching for growth promoters to compensate for this gap. There are many growth promoters as antibiotic growth promoters (AGPs). AGPs are widely used to prevent poultry disease through prevention of pathogens and improve growth performance. But the use of AGPs in the diet of the poultry can cause serious problems such as antibiotic-resistant pathogens and drug residues [1]. Also, Tayeri et al. [2] reported routine use of an antibiotic (flavomycin) increase Enterococci. Thus, searching for alternatives to antibiotics is very urgent. Prebiotics are used as one of the alternatives to antibiotic growth promoters [3]. Prebiotics are non-digestible components of feed derived from sugars, including raffinose, galactooligosaccharides, and β-glucans [4]. They prevent enteric diseases and improve performance in poultry. Prebiotics have been shown to alter the immune system, modify gastrointestinal microflora, and reduce invasion of the pathogen such as *Salmonella spp.* and *E. coli* [5]**.** The major function of prebiotics is to activate the metabolism of some groups of beneficial bacteria in the intestinal tract and/or stimulate their growth. Pelicano et al. [6], Spring et al. [7]**,** and Xu et al. [8] have shown that the addition of prebiotics to broilers’ diet results in the improvement of the gut microflora and growth as well**.** The gut microflora composition plays an important role in digestion, which can be performed in a positive, negative, or neutral manner. Gastrointestinal microflora modifications reduce attachment of the pathogen and may have a beneficial effect on the nutrients digestibility [9]**.** Administration of agrimos® (Mannan-oligosaccharides (MOS), and β-Glucans), which obtained from a specific strain of *Saccharomyces cerevisiae* cell wall was found to improve the productive performance and immune functions in broiler chickens [10]**.** Also, Wang et al. [1] and Dawood et al. [11] reported the antioxidant effect of Mannan-oligosaccharides in broilers and red sea bream, respectively.

Hence, this study was conducted to examined agrimos® (MOS and β-Glucans) effect on growth, immunity, serum biochemistry, histopathology, selected liver gene expressions, and colonization of *E. coli* in broilers.

## Results

### Clinical signs and postmortem (PM) lesions of *E. coli* infection

Experimental infection with *E. coli* revealed suggestive clinical signs and PM lesions after 48 h. post-infection in the form of depression with whitish diarrhea.

PM lesions revealed liver enlarged and congested and distended gallbladder and cecum. Such changes were less prominent in the agrimos-infected group (see Table 1).

**Table 1:** The mortality rate of broiler chicken infected with *E. coli* and fed on agrimos**®** at 35 days (*n* = 30)

Groups Parameter	Control noninfected	Control infected	Agrimos® noninfected	Agrimos® infected
Total No.	30	30	30	30
Dead No.	1	2	0	1
Survival %	96.67	93.33	100	96.67
Mortality %	3.33	6.67	0	3.33

### Growth performance

The feed supplemented with agrimos® in the third and fourth groups significantly (*P* ≤ 0.05) increased the body weight (BW) and total gain along with the whole experiment, and improved feed conversion ratio (FCR) compared to the other groups (see Table 2). On the other hand, the above-mentioned parameters were decreased significantly (*P* ≤ 0.05) in the control-infected group as compared to the other groups.

**Table 2:** Growth performance of broiler chicken infected with *E. coli* and fed on agrimos**®** at 35 days (*n* = 30)

Groups Parameters	Control noninfected	Control infected	Agrimos® noninfected	Agrimos® infected
Initial body weight (g/chick)	45.33 ± 0.88^a^	45 ± 1.00^a^	45.67 ± 0.88^a^	45.67 ± 1.20^a^
Final body weight (g/bird)	1544.33 ± 1.76^b^	1358.67 ± 2.03^c^	1736.33 ± 2.33^a^	1547.00 ± 1.73^b^
Total Weight gain (g/bird)	1499 ± 1.53^b^	1313.67 ± 2.19^c^	1690.67 ± 1.45^a^	1501.33 ± 0.67^b^
FCR value	1.67 ± 0.005^b^	1.87 ± 0.006^a^	1.54 ± 0.003^c^	1.66 ± 0.003^b^

Values are expressed as mean ± standard errors. Means in the same row (a-c) with different letters significantly differ at (*p* ≤ 0.05)

### Serum parameters related to liver function

In (Table 3), there were significant (*P* ≤ 0.05) increased in the activities of alanine transaminase (ALT) and aspartate aminotransferase (AST) enzymes in the group of broiler chickens infected with *E. coli* compared to the control non-infected group. However, serum total protein and albumin were significantly (*P* ≤ 0.05) decreased. Agrimos® supplementation did not affect serum ALT, AST, and albumin, but the serum total protein and globulin affected significantly (*P* ≤ 0.05) when compared with the control non-infected group.

**Table 3:** Serum liver function of broiler chicken infected with *E. coli* and fed on agrimos**®** at 35 days (*n* = 5)

Groups Parameters	Control noninfected	Control infected	Agrimos® noninfected	Agrimos® infected
ALT (u/l)	19.33 ± 0.88^b^	26.33 ± 0.88^a^	18.67 ± 0.88^b^	20.67 ± 0.67^b^
AST (u/l)	53.33 ± 1.76^b^	92.67 ± 0.88^a^	51.33 ± 0.88^b^	52.67 ± 1.45^b^
Total protein (g/dl)	2.27 ± 0.08^b^	1.9 ± 0.06^c^	2.9 ± 0.12^a^	2.7 ± 0.11^a^
Albumin (g/dl)	1.20 ± 0.02^a^	0.47 ± 0.05^b^	1.24 ± 0.03^a^	1.21 ± 0.01^a^
Globulin (g/dl)	1.06 ± 0.09^b^	1.43 ± 0.01^a^	1.66 ± 0.12^a^	1.49 ± 0.11^a^

Values are expressed as mean ± standard errors. Means in the same row (a-c) with different letters significantly differ at (*p* ≤ 0.05)

### Serum antioxidant enzymes activity

Serum MDA level was significantly (*P* ≤ 0.05) increased in broiler chickens infected with *E. coli* (see Table 4). However, serum catalase (CAT) and superoxide dismutase (SOD) activities were significantly (*P* ≤ 0.05) reduced compared to the other groups. Agrimos® supplementation significantly (*P* ≤ 0.05) decreased serum lipid peroxidation (MDA) with significant (*P* ≤ 0.05) increased serum CAT and SOD when compared to the control-infected group.

**Table 4:** Serum antioxidant of broiler chicken infected with *E. coli* and fed on agrimos**®** at 35 days (*n* = 5)

Groups Parameters	Control noninfected	Control infected	Agrimos® noninfected	Agrimos® infected
MDA (n.mol/l)	0.65 ± 0.02^c^	1.17 ± 0.08^a^	0.65 ± 0.01^c^	0.8 ± 0.03^b^
CAT (u/ml)	12.7 ± 0.01^b^	9.73 ± 0.15^c^	15.36 ± 0.13^a^	13.03 ± 0.09^b^
SOD (u/ml)	12.18 ± 0.0^c^	9.37 ± 0.0^d^	17.2 ± 0.89^a^	15.0 ± 0.0^b^

Values are expressed as mean ± standard errors. Means in the same row (a-c) with different letters significantly differ at (*p* ≤ 0.05)

### Immune response against Newcastle disease (ND)

Immune response to vaccination of ND, which evaluated by Hemagglutination Inhibition (HI) titer, revealed a difference in log2 of GM. There was a significant difference in HI titer in the second week after infection between each of the *E. coli* infected group and the control negative group, and the agrimos infected group. In addition, the agrimos group significantly differs about the other three groups.

After vaccination in the second and third week, this pattern was observed where no difference was recorded between each of the *E. coli* infected group, control non-infected, and agrimos infected group. Besides, the agrimos group significantly differs about the other three groups (see Table 5).

**Table 5:** HI titer for ND at different periods (*n* = 5)

Groups Age	Control infected	Control noninfected	Agrimos® infected	Agrimos® noninfected
**0 day**	**4.6 ± 0.07**^**b**^ **C.V = 11.6%**	**4.8 ± 0.06**^**b**^ **C.V = 9.03%**	**4.8 ± 0.06**^**b**^ **CV = 9.3%**	**5.2 ± 0.06**^**a**^ **CV = 8.3%**
**1st weeks p. v**	**5.2 ± 0.06**^**c**^ **C.V = 8.6%**	**5.6 ± 0.7**^**b**^ **CV = 9.5%**	**5.6 ± 0.07**^**b**^ **CV = 9.5%**	**6.2 ± 0.06**^**a**^ **CV = 7.2%**
**2nd weeks P. v**	**6 ± 0.09**^**b**^ **C.V = 11.5**	**6.2 ± 0.11**^**b**^ **CV = 13.4**	**6.2 ± 0.06**^**b**^ **CV = 6.9%**	**7 ± 0.09**^**a**^ **CV = 10%**
**3rd weeks P. v**	**4.95 ± 0.09**^**b**^ **C.V = 14.5%**	**4.6 ± 0.24**^**b**^ **CV = 11.6%**	**4.8 ± 0.06**^**b**^ **CV = 9%**	**6 ± 0.09**^**a**^ **CV = 11.7%**
**Average of log**_**2**_**GM ± SEM**	**5.19 ± 0.3**^**b**^	**5.3 ± 0.37**^**b**^	**5.35 ± 0.34**^**b**^	**6.1 ± 0.37**^**a**^

No difference between item carries the same letter in the same row

*GM* Geometric mean, *CV* Coefficient of variation, *p. v* Post vaccination

### Colonization of *E. coli*

The colonization of *E. coli* in the control infected group in different organs showed several rates of 67, 44, 22, 44, and 53% in the respiratory tract, liver, gallbladder, spleen, and fecal swab, respectively (see Table 6). However, in the agrimos infected group, the colonization of *E. coli* in different organs showed reduced rates of 22, 33, 22, 11, and 33% in the respiratory tract, liver, gallbladder, spleen, and fecal swab, respectively**.**

**Table 6:** Colonization of *E. coli* and rate of shedding as judged by intestinal colonization (*n* = 5)

	R. T. swab			Liver			G. bladder			Spleen			Fecal swab		
	+	T	%	+	T	%	+	T	%	+	T	%	+	T	%
*E. coli*	6	9	67	4	9	44	2	9	22	4	9	44	8	15	53
*E. coli* + A^a^	2	9	22	3	9	33	2	9	22	1	9	11	5	15	33

*R. T. swab* Respiratory tract swab ^a^*E. coli* + A = *E. coli + Agrimos***®**

### The weight of immune organs

**Fig. 1:**
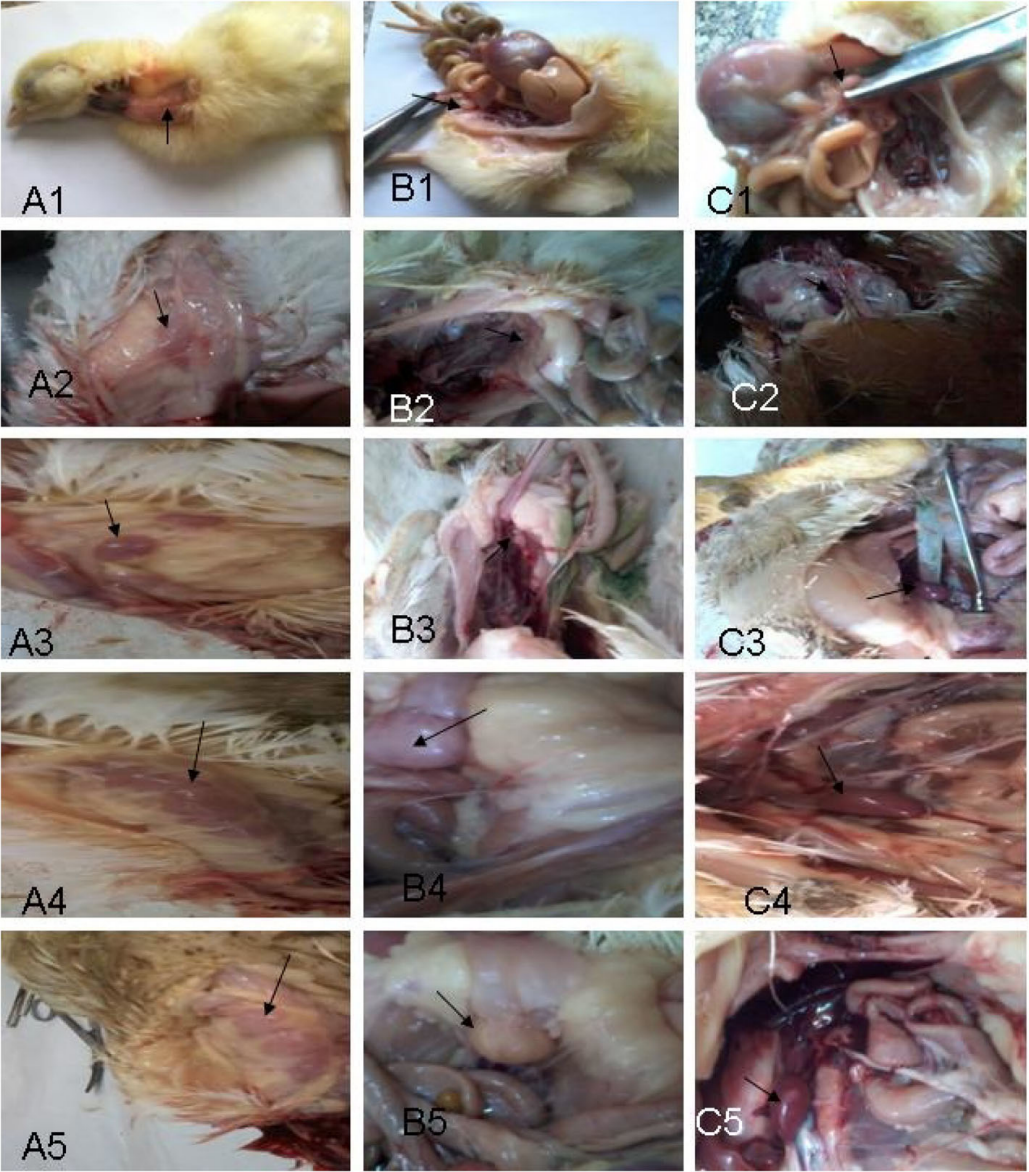
Thymus of one-day-old chicken **(**A1). Thymus of control non-infected group at 35 days (A2). Thymus of control infected group at 35 days (A3). Thymus of agrimos non-infected group at 35 days (A4). Thymus of agrimos infected group at 35 days (A5). Bursa of one-day-old chicken **(**B1). Bursa of control non-infected group at 35 days (B2). Bursa of control infected group at 35 days (B3). Bursa of agrimos non-infected group at 35 days (B4). Bursa of agrimos infected group at 35 days (B5). Spleen of one-day-old chicken **(**C 1). Spleen of control non-infected group at 35 days (C 2). Spleen of control infected group at 35 days (C 3). Spleen of agrimos non-infected group at 35 days (C 4). Spleen of agrimos infected group at 35 days (C 5)

At zero, 15, and 35 days of age, the weight of the immune organ versus body weight was evaluated (see Table 7 and Fig. 1)**.** On days 15 and 35, the results of thymus weight showed a significant increase (*P* ≤ 0.05) in the agrimos non-infected and infected groups in comparison with the other groups. However, there was a significant decrease (*P* ≤ 0.05) in the control infected group compared to the control non-infected group.

**Table 7:** The weight of Immune organs of broilers at 35 days (*n* = 5)

Organs	Treatment and age											
	C-			C+			A-			A+		
	Zero- day	At 15 days	At 35 days	Zero-day	At 15 days	At 35 days	Zero- day	At 15 days	At 35 days	Zero- day	At 15 days	At 35 days
**T/B.W.**	0.287 ± 0.033^Y^	0.59 ± 0.012 ^bX^	0.134 ± 0.008 ^BZ^	0.287 ± 0.033^Y^	0.377 ± 0.013 ^cX^	0.069 ± 0.006 ^CZ^	0.287 ± 0.033^Y^	0.65 ± 0.006^aX^	0.282 ± 0.012^AY^	0.287 ± 0.033^Y^	0.627 ± 0.009 ^aX^	0.276 ± 0.011 ^AY^
**S/B.W.**	0.036 ± 0.007^Y^	0.046 ± 0.003 ^dY^	0.132 ± 0.011 ^BX^	0.036 ± 0.007^Z^	0.094 ± 0.004 ^cX^	0.056 ± 0.006 ^CY^	0.036 ± 0.007^Z^	0.11 ± 0.006 ^bY^	0.229 ± 0.009 ^AX^	0.036 ± 0.007^Z^	0.127 ± 0.003 ^aY^	0.159 ± 0.005 ^BX^
**B/B.W.**	0.157 ± 0.024^Y^	0.329 ± 0.003 ^bX^	0.121 ± 0.004 ^CY^	0.157 ± 0.024^Y^	0.259 ± 0.01 ^cX^	0.064 ± 0.006 ^DZ^	0.157 ± 0.024^Y^	0.394 ± 0.003 ^aX^	0.355 ± 0.014 ^AX^	0.157 ± 0.024^Y^	0.338 ± 0.017 ^bX^	0.317 ± 0.013 ^BX^

Values are expressed as mean ± standard errors. Means in the same row (a-d), (A-C) and (X-Z) with different letters significantly differ at (*p* ≤ 0.05)

*T* Thymus *B. W*. Body weight, *S* Spleen, *B* Bursa

The results of spleen on day 15 showed that there was a significant increase (*P* ≤ 0.05) in the agrimos infected group compared to the other groups. Furthermore, there was a significant increase (*P* ≤ 0.05) in the agrimos non-infected group compared to the control non-infected and infected groups. However, on day 35, the agrimos non-infected group witnessed a significant increase in the spleen’s weight (*P* ≤ 0.05) compared to the other groups. As well, the control infected group had a significant increase (*P* ≤ 0.05) compared to the control non-infected group on day 15 but a significant decrease (*P* ≤ 0.05) on day 35.

The bursa’s weight was significantly increased (*P* ≤ 0.05) in the agrimos non-infected group compared to the other groups, but it was significantly decreased (*P* ≤ 0.05) in the control infected group compared to the other groups on days 15 and 35.

The thymus’ and bursa’s weight in the control non-infected group were significantly (*P* ≤ 0.05) decreased, but the spleen’s weight was significantly (*P* ≤ 0.05) increased. The control-infected group showed a significant (*P* ≤ 0.05) increase in the weights of the thymus, spleen, and bursa on day 15, and then showed a significant (*P* ≤ 0.05) decrease on day 35. The agrimos non-infected and infected groups showed a significant (*P* ≤ 0.05) increase in the weights of the spleen and bursa on days 15 and 35 but showed a significant (*P* ≤ 0.05) increase in the weight of the thymus on day 15 only, which returned to its normal size on day 35.

### The pathological findings of broiler chickens

**Fig. 2:**
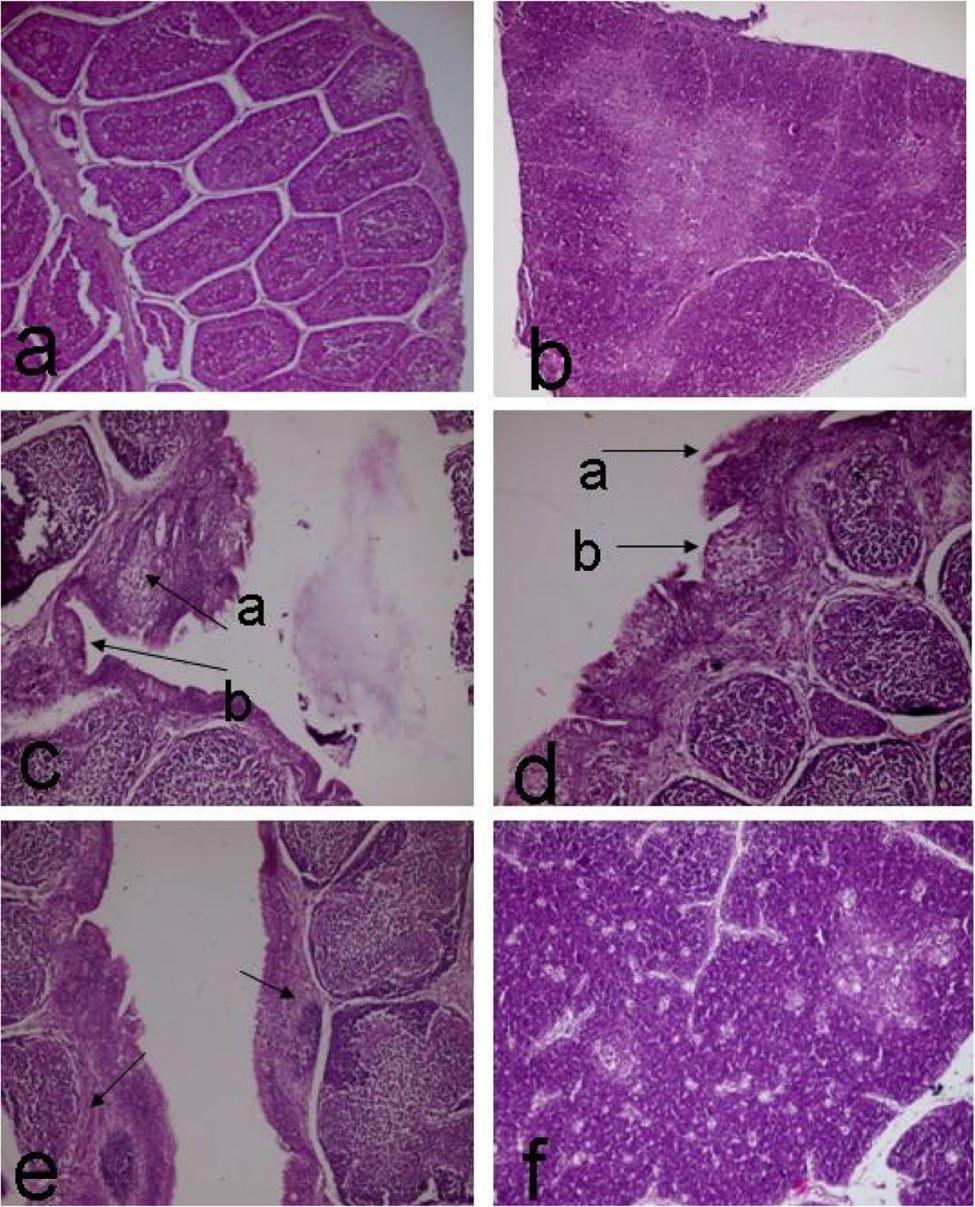
**a**. showing normal bursa of Fabricius. Control negative one-day-old chick. **b**. showing normal thymus. Control negative one-day-old chick. *C. bursa* of Fabricius showing variable degree of epithelial hyperplasia (arrow a) and ulceration (arrow b). Control infected group at 15 days post infection. **d**. Bursa of Fabricius showing variable degree of epithelial hyperplasia (arrow a) associated with degeneration and ulceration (arrow b). Control infected group at 15 days post infection. **e**. Bursa of Fabricius showing variable degree of epithelial hyperplasia associated with degeneration and sub cortical fibrous tissues proliferation. Control infected group at 15 days post infection. **f**. Thymus showing numerous holes on the cortex and medulla. Control infected group at 15 days post infection. H&E. a and b X 100 other Figs. X 200

**Fig. 3:**
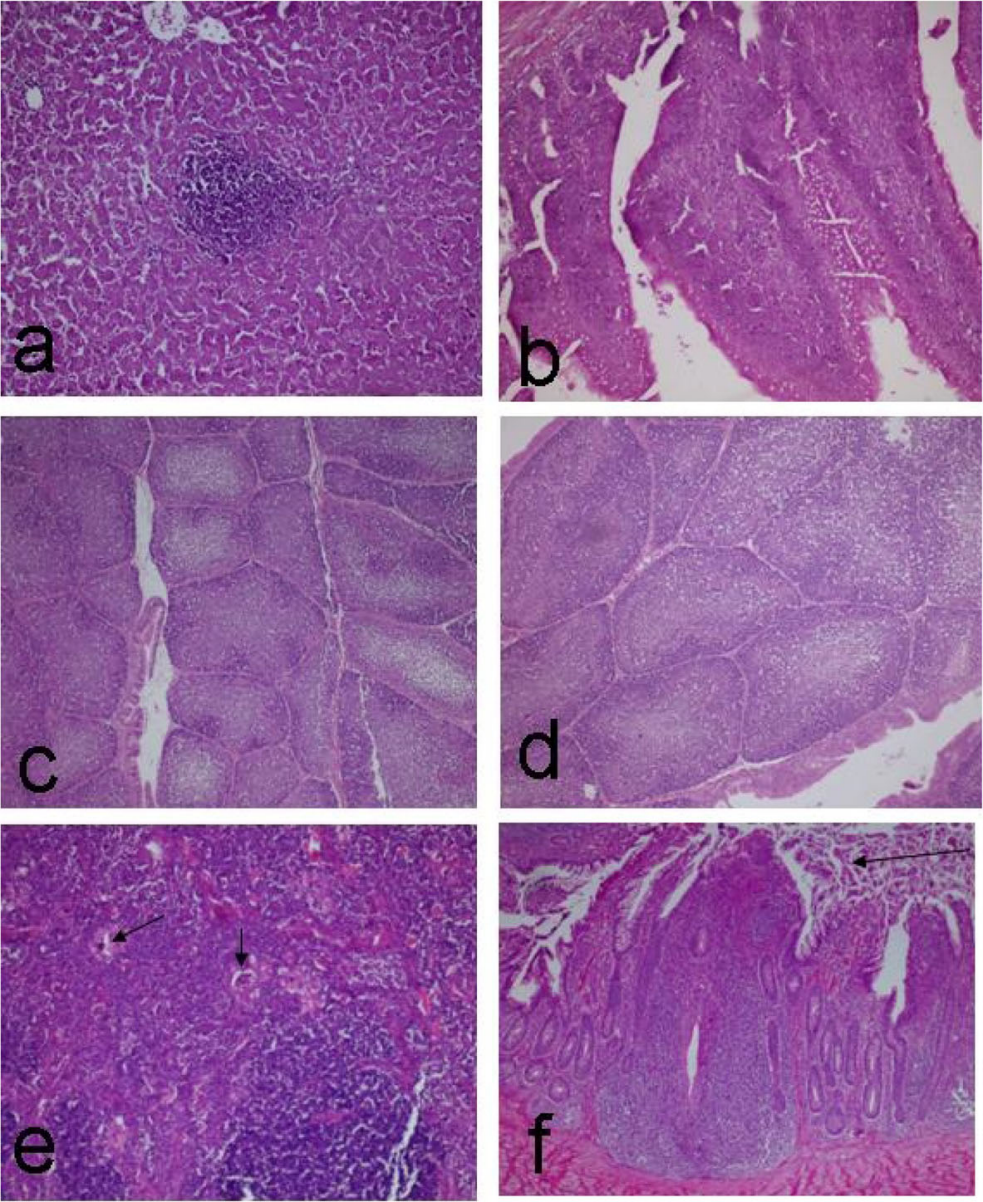
**a**. Liver showing focal area of round cells aggregation. Control infected group at 35 days post infection. **b**. Intestine (duodenum) showing hyperplasia of the epithelial lining the intestinal villi associated with vaculation. Control infected group at 35 days. ***C****. bursa* of Fabricius showing normal atypical fold. Agrimos**®** non-infected group at 15 days. **d**. Bursa of Fabricius showing pores within the cortex. Agrimos**®** non-infected group at 35 days. **e**. Thymus showing thrombus formation (arrow). Agrimos**®** non-infected group at 15–35 days. **f**. Caecal tonsil showing epithelial sloughing (arrow). Agrimos**®** non-infected group at 15–35 days. H&E. X 200 for all figures except c X 100

The histopathological examination of the spleen, bursa, thymus, liver, and duodenum of the chicks of the control non-infected group (on days 15 and 35 after infection), showed no obvious histopathological alterations (see Fig. 2a and b). Concerning the control infected group (on day 15), the bursa showed variable degrees of epithelial hyperplasia, degeneration, and ulceration in some cases. These changes were associated with subcortical fibrous tissue proliferation in some cases (see Fig. 2c, d, and e). The thymus of this group showed a narrowing in the cortical width and an increase in the medulla in most cases. Some other cases had the appearance of clear areas or holes that contained small dark nuclei (a defining characteristic of the apoptosis in the lymphoid organ), (see Fig. 2f). No microscopical changes were detected on the spleen of this group. Regarding the control infected group (on day 35), similar changes were seen on day 15, but more holes were detected in the thymus. Also, the focal area of round cells aggregation was seen in the liver of this group (see Fig. 3a). The duodenum of this group showed hyperplasia on the epithelial cells lining the intestinal villi accompanied by vacuolation (see Fig. 3b).

**Fig. 4:**
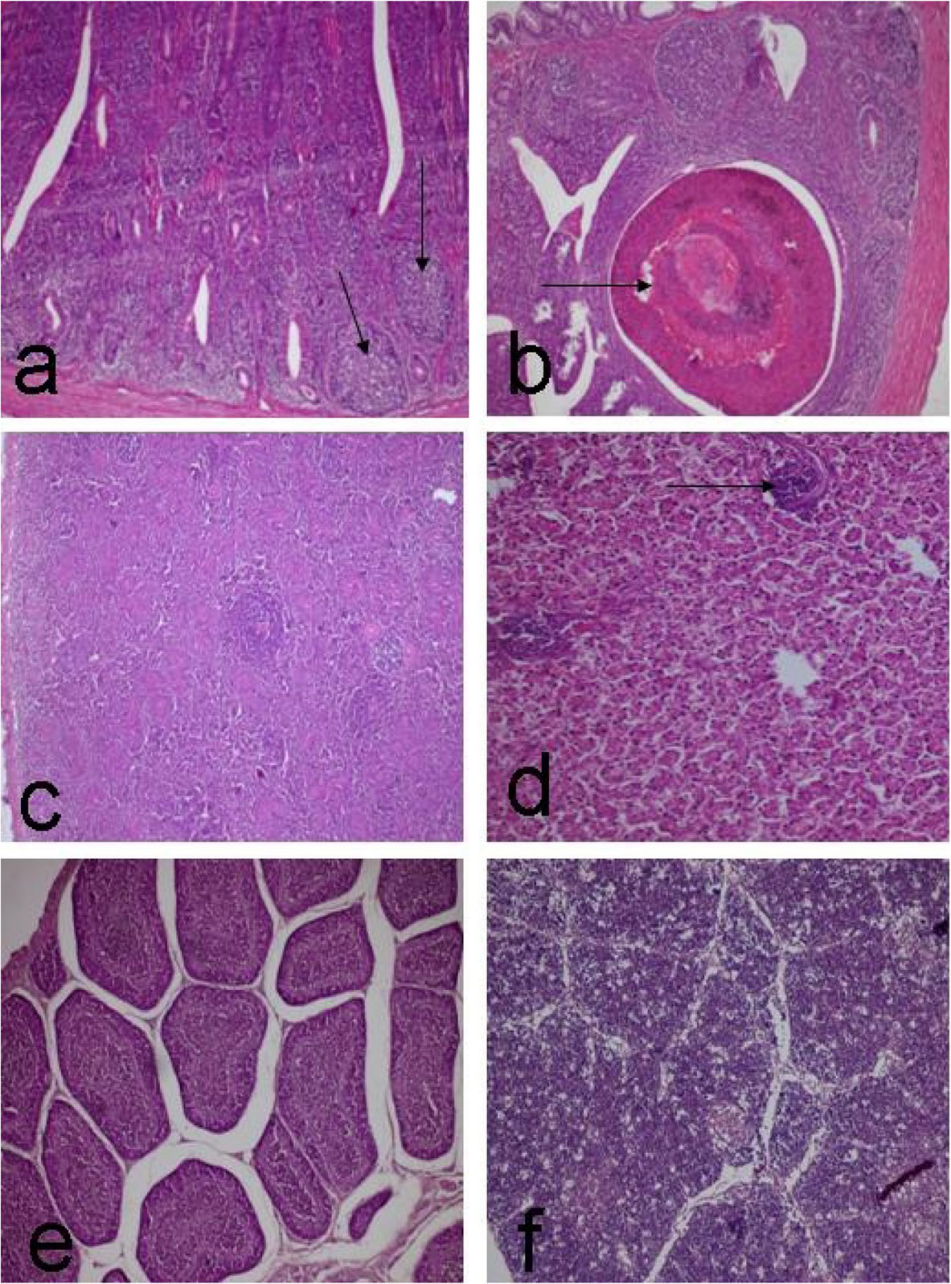
**a**. Caecal tonsil showing normal lymphoid aggregation (arrow). Agrimos**®** non-infected group at 15–35 days. **b**. Caecal tonsil showing necrotic cyst (arrow). Agrimos**®** non-infected group at 15–35 days. **c**. Spleen showing normal appearance. Agrimos**®** non-infected group at 15–35 days. **d**. Liver showing mild focal areas of round cells aggregation (arrow). Agrimos**®** non-infected group at 15–35 days. **e**. Bursa of Fabricius showing inter follicular edema. Agrimos**®** infected group at 15 days post infection. **f**. Thymus showing numerous cortical holes in cortex and medulla (these holes containing apoptotic bodies). Agrimos**®** infected group at 15 days post infection. H&E. X 200 for all figures except e X 100

The histopathological examination of the bursa of Fabricius in most cases in the agrimos non-infected group on day 15 (4 out of 5), showed normal a typical fold (plica) (see Fig. 3c). Some of these cases showed a highly dilated germinal center and narrow cortex, associated with a mild degree of epithelial lobulation. One case showed follicular lymphocytic depletion. Similar histopathological changes were seen on the bursa of Fabricius in the agrimos non-infected group on day 35. One case showed pores within the cortex of many plicae (see Fig. 3d), and this was associated with interfollicular edema. Regarding the thymus gland in both times (days 15 and 35), no obvious histopathological alterations were seen on the thymic lobe except in few cases (2 out of 10), which showed thrombus formation within the thymic vasculature (see Fig. 3e). Concerning the caecal tonsil of the agrimos non-infected group on days 15 and 35, just epithelial sloughing was seen in some cases (see Fig. 3f), and no obvious histopathological alterations were viewed on the lymphoid nodules (lymphoid aggregation), (see Fig. 4a). Two cases (out of 10) showed a necrotic cyst (see Fig. 4b). No obvious microscopical changes were seen on the spleen of this group (see Fig. 4c). The microscopical examination of the liver of this group showed mild focal areas of round cell aggregation (see Fig. 4d) in two cases (out of 10).

The histopathological examination of the bursa of Fabricius in most cases in the agrimos infected group on days 15 and 35 after infection (2 out of 4), showed normal morphological appearances but 2 cases showed interfollicular edema (see Fig. 4e) and epithelial hyperplasia and folding associated with mucous (goblet) cell activation. Regarding the thymus gland in both times (days 15 and 35), no obvious histopathological alterations were seen on the thymic lobe except in few cases (1 out of 4), which showed focal areas of necrosis and hyalinization (see Fig. 4f). No obvious microscopical changes were seen on the spleen of this group (see Tables 8 and 9).

**Table 8:** Histopathological assessment of dietary agrimos**®** supplementation on *E. coli* challenged broiler chicken ((*n* = 10)

Lesions	Infected group						Agrimos non-infected group						Agrimos infected group					
	At 15 days			At 35 days			At 15 days			At 35 days			At 15 days			At 35 days		
	B	T	S	B	T	S	B	T	S	B	T	S	B	T	S	B	T	S
Sub capsular heterophilic aggregation	–		Normal	–		Normal	Normal	Normal	Normal	Normal	Normal	Normal	–	Normal	Normal	–		Normal
Sub and interstitial edema	–			–									+			+		
Interstitial hemorrhages	–			–									–			+		
Interstitial connective tissue proliferation	–			–									–			–		
Epithelial folding and lobulation	+			–									–			–		
Epithelial hyperplasia	+			+									+			+		
Epithelial degeneration and/or necrosis	+			+									–			+		
Reduction in size and numbers of follicles	–			–									–			–		
Follicular atrophy or cortical atrophy	–			–	+								–			+	+	
Follicular cyst and necrosis	–			–									–			+		
Lymphocytic depletion	–			–									–			+		
Appearance of holes in medulla	–	+		–	++								–			–		
Reticular cells proliferation	–			–									–			–		
Vasculitis	–			–									–			–		
Cortical necrosis	–			–									–			–		
Apoptotic bodies and/or karyorhexis	–	+		–									–			–		
Hyalinization and thrombosis of blood vessels	–			–									–			–		

+++ Sever ++ Moderate + Mild B = Bursa T = Thymus S=Spleen

**Table 9:** Incidence of pathological changes in organs at different periods (*n* = 10)

Groups	Infected group								Agrimos non-infected group								Agrimos infected group							
Periods	At 15 days				At 35 days				At 15 days				At 35 days				At 15 days				At 35 days			
Parameter	N	A	T	%	N	A	T	%	N	A	T	%	N	A	T	%	N	A	T	%	N	A	T	%
B	2	2	4	50	1	3	4	75	4	1	5	20	4	1	5	20	2	2	4	50	2	2	4	50
T	4	0	4	0	2	2	4	50	3	2	5	40	4	1	5	20	3	1	4	25	3	1	4	25
S	3	1	4	25	4	0	4	0	5	0	5	0	5	0	5	0	4	0	4	0	4	0	4	0

*B* Bursa *T* Thymus *S* Spleen, *N* Normal *A* Abnormal *T* Total % = Ratio

### Gene expression related to *E. coli* infection and Agrimos supplementation

**Fig. 5:**
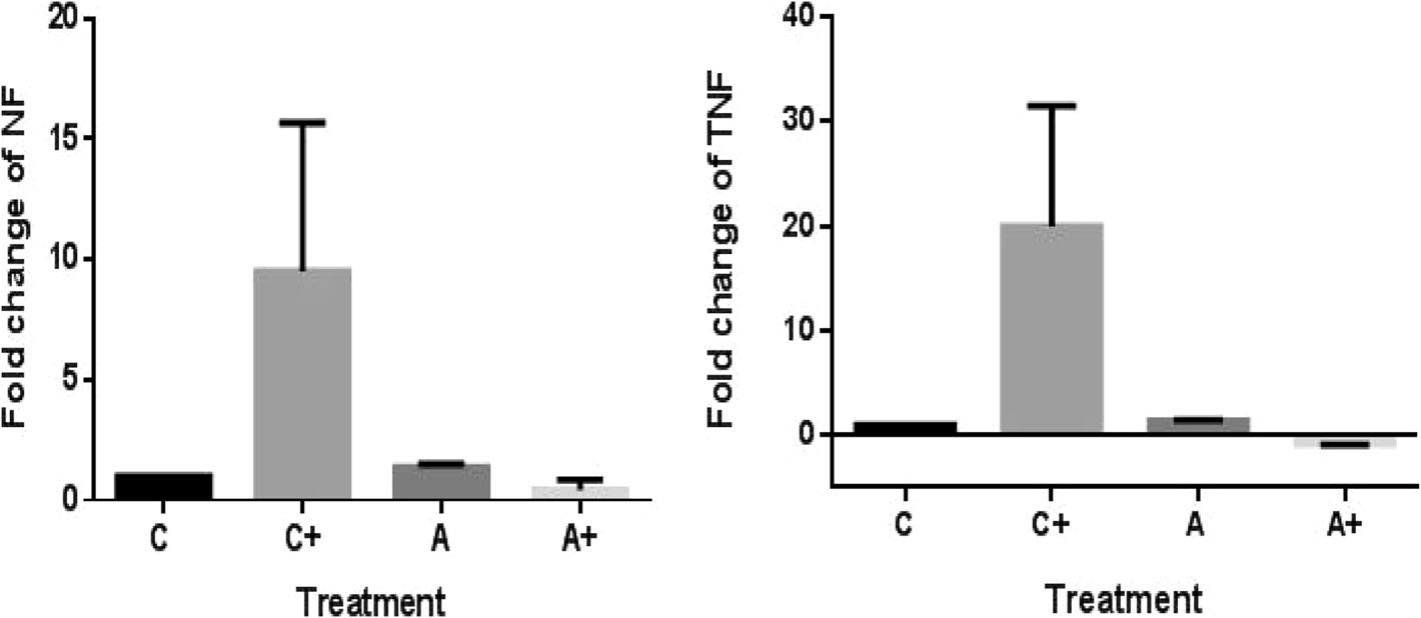
Changes in the gene expression of NF (right) and TNF-α (left) among experimental groups. C (negative control), C+ (positive control), A (agrimos**®** treated without infection), A+ (agrimos**®** treated group with *E. coli* infection)

The results of Q-PCR revealed that the expression levels of nuclear factor-kappa (NF) and tumor necrosis factor-alpha (TNF) genes of the liver tissues were significantly (*P* ≥ 0.05) increased in the group infected by *E. coli* (Fig. 5) compared to the negative control group.

In the agrimos non-infected group, the gene expression of NF and TNF in liver tissues did not show significant differences in comparison with the control non-infected group. The levels of such genes were similar to those of the control non-infected group.

It was found that the NF and TNF expression levels were significantly (*P* ≥ 0.05) reduced due to agrimos® supplementation when compared to those of the infected group without supplementation. This shows the protective effect of agrimos® supplementation on the expression of the above-mentioned genes of the infected broiler chicks.

## Discussion

In the present study, the control infected group with *E. coli* showed varied clinical signs according to their age, an organ involved, and concurrent disease conditions. Results of clinical signs in the *E. coli* infected groups (even in the treated one) similar to the results of Remus et al. [12] who recorded severe liver damage occurs in cases of *E. coli* infection. However, dietary supplementation of agrimos**®** decreased the clinical signs and PM lesions. This result is consistent with Sohail et al. [13]. On the other hand, the results of the growth performance in the control infected group showed significant retardation. This retardation may be attributed to the *E. coli* infection. This result is compatible with Manafi et al. [14]**.** Meanwhile, agrimos**®** dietary supplementation significantly improved growth performance, which may be ascribed to the increased digestibility and feed intake as well. Jahanian and Ashnagar [15] reported that dietary MOS supplementation improves digestibility coefficients of dry matter and crude protein. These results in harmony with the results of El-Far et al**.** [16] and Mousa et al. [17] in broilers and Japanese quail, respectively.

Results of biochemical parameters showed a significant increase in serum AST and ALT activities, and globulin concentration, meanwhile, serum total protein and albumin were significantly decreased in the control infected group. These results agree with Manafi et al. [14], who reported the same results when birds were challenged with *E. coli*. These results may be attributed to *E. coli* infection. Pathogenic *E. coli* infection in broilers leads to local or systemic infection (colibacillosis). Colibacillosis is an infectious disease characterized by acute fatal septicemia or sub-acute fibrinous pericarditis, airsacculitis, salpingitis, and peritonitis [18]. In the present study, *E. coli* infection occurred colibacillosis infection, which indicated by results of *E. coli* colonization and increased serum biochemical parameters. In addition, Sharma et al. [19] reported that the increase in serum activities of ALT is indicative of cellular injury to hepatocytes and AST in cardiac muscles and hepatocytes as well. On the other hand, hypoproteinemia may be due to hepatocyte damage, which results in failure in the synthesis of plasma protein, where the liver is a site for albumin synthesis. Also, hypoproteinemia may be due to kidney disease, which leads to protein loss and congestive heart failure [19, 20]. At the same time, hyperglobulinemia is associated with liver cirrhosis, hepatitis, and Kuffer cell proliferation [19]. However, dietary supplementation of agrimos**®** not affected serum AST and ALT activity and albumin concentration but significantly increased total protein and globulin of the serum. The above results are consonant with those of Mousa et al. [17]. Meanwhile, Yalçinkaya et al. [21] found a significant decrease in the activities of AST and ALT. The results of the present study are incompatible with Rokade et al. [22], who found a significant increase in the serum activities of AST and ALT, and protein concentration in broilers dietary supplemented with MOS. These results may be attributed to the antioxidant properties of the MOS, which was reported by Dawood et al. [11]. On the other hand, results of antioxidant enzymes and MDA in harmony with the results of Zhou et al. [23] found a significant decrease of antioxidant enzymes in birds infected with necrotic enteritis. These results may be attributed to infection, which was previously reported by Mishra B, Jha [24]. On the other hand, Abudabos et al. [25] reported that *Salmonella typhimurium* had no impact on total antioxidant capacity or oxidative stress in challenged broilers. However, agrimos**®** supplementation significantly decreased serum MDA and increased CAT. This result is compatible with that of El-Kader et al. [26] in broilers.

The reduction of HI titer in the *E. coli* infected groups may be attributed to the stress of infection and diarrhea, which may change the acid-base balance [27]. In addition, the increase in HI titer due to the nutrition of agrimos® appeared to be insignificant. This may be attributed to the fact that agrimos® tend to initiate non-specific immunity rather than humoral immunity [28]. The dietary MOS stimulates humoral immune responses against infectious bursal disease (IBDV) and Newcastle disease (NDV) vaccine viruses [29]. Regarding results of *E. coli* colonization, the higher colonization rates of *E. coli* in different organs of control infected group were in the respiratory tract, fecal swab, liver, and spleen, where avian pathogenic *E. coli* cause severe respiratory and systemic diseases in poultry [30]. This result is in harmony with that of Manafi et al. [14]. However**,** this colonization was decreased by agrimos**®** dietary supplementation, which was supported by the results of Sohail et al. [13].

Cloacae bursa plays a crucial role in the poultry immune system. In addition, the bursal weight reflects the anatomical response to immune status in broilers [14]. In the present investigation, there were significantly decreased in thymus and bursa’s weights versus body weight in the control infected group, which was supported by Gottardo et al. [31]. However, these results are incompatible with those obtained by Manafi et al. [14]. Also, the spleen’s weight versus body weight in the control infected group significantly increased on day 15 then decreased on day 35. These results may be attributable to the infection, which was supported by the results of Huff et al. [32] in turkeys. On the other hand, there were significantly increased in thymus’, spleen, and bursa’s weights versus body weight in the agrimos**®** groups. These results are in harmony with that of Sohail et al. [13].

Malfunctions of lymphoid organs reduce resistance to bacterial, viral, parasitic, and fungal infections. Thus birds become more susceptible to other infections, leading to high economic losses due to morbidity and mortality [33]. Therefore, the rational assessment of the immunosuppression in the poultry warrants rapid and accurate diagnostic approaches. Thus, the present study focused on the pathology of lymphoid organs.

The pathological changes in the control infected group were apoptosis in the lymphoid organ and focal area of round cell aggregation in the liver. These results are in harmony with that of Kumar et al. [34]. Meanwhile, spleen revealed depletion of lymphocytes, areas of congestion, and necrosis of lymphocytes from the white pulp. Also, bursa of Fabricius showed lymphoid depletion in follicles, and thymus showed some lesions like medullary congestion and mild cortical depletion [33]. Remus et al. [12] reported *E. coli* caused severe liver and epithelial damage. However, agrimos**®** supplementation to broilers revealed no pathological changes. This result is compatible with that of Awaad et al. [10]. As well, in the infected group, the supplementation of agrimos**®** mitigated the pathological changes caused by infection. This result may be due to the composition of agrimos**®**, which contains β-Glucans and MOS.

Pro-inflammatory cytokines such as nuclear factor (NF) and tumor necrosis factor (TNF) produced during the acute phase response can induce the production of several acute-phase proteins (APPs) by hepatocytes [35]. In the present study, it was found that agrimos**®** had no clear effect on liver NF or TNF expression levels of the uninfected broiler chicks. However, the reduction in liver enzyme activities and the down-regulation of NF and TNF gene expressions indicated the protective effects and anti-inflammatory properties of agrimos**®** in broiler chicks with *E. coli* infection. These results are consistent with that obtained by Markazi [36] in *Salmonella* challenge broilers.

Most of the previous research has been performed on healthy chicks without infection conditions or has lacked to clear some gene expressions at the site of infection. *E. coli* infection is considered a common disease in poultry production, which can induce different oxidative and inflammatory stress. Thus, in the present study, *E. coli*-inoculated chickens were used to investigate the effects of agrimos**®** on healthy chickens and under such conditions.

Together with TNF or NF data, agrimos**®** can apparently regulate the cytokine responses of broiler chicks with *E. coli* infection, and its immunomodulatory effects seem to be protective by repressing the ongoing inflammation. In brief, the decreased TNF and NF expressions of liver tissues in the agrimos**®** fed broiler chicks with *E. coli* infection may be an indication of slowdown inflammation and gradual restoration of the infected broiler chicks’ health status.

### Socio-economic importance

Veterinary medicine plays a crucial role in public health. The scope of veterinary public health includes food hygiene practices development and supervision in addition to zoonotic disease control and eradication [37]. In addition, antibiotic usage in animals plays a major role in the emerging public health crisis of antibiotic resistance [38]. Thus, nowadays, using probiotics or prebiotics have become popular. They are considered an alternative to Antibiotic Growth Promoters (AGP). Probiotics are supplemented into animal feed for improving growth performance along with preventing and controlling enteric pathogens [39].

Attention should be given to the enteric pathogens in the poultry field due to their economic effects (death, loss of weight, and poor conversion ratio). Besides, the spread of *E. coli* and the high usage of antibiotics in the poultry industry can result in bacterial resistance to such antibiotics. Instead, it is recommended to use prebiotics.

## Conclusion

In the present study, the control infected group showed retardation in the growth performance parameters with abnormal serum biochemical parameters. These retardation and abnormalities were mitigated by agrimos**®** supplementation, which improved growth performance. Also, agrimos**®** improved the non-specific immunity, which in turn led to an improvement in the specific immunity against ND, which was reported in the discussion above. On the other hand, agrimos® reduced oxidative stress and slowed down inflammation that occurs as a result of *E. coli* infection through decreased TNF and NF expressions in liver tissue. Consequently, there was no need to used antibiotics. So, Agrimos® proved to be a good alternative to antibiotics for controlling and reducing *E. coli* infection and colonization.

## Methods

### Agrimos®

The combination of Manno-oligosaccharides (MOS), and Beta-Glucans, which was extracted from *Saccharomyces cerevisiae* with a specific dedicated manufacturing process produced agrimos**®**. Agrimos**®** modulates the non-specific immune response and favors the development of the specific response. It was made in Lallemand Company (a Canadian company) and imported by Egavet (Egyptian Group for Trade and veterinary Services) Company, Egypt.

### Experimental design, feeding program, and management

125 mixed-sex chicks (one-day-old), were used (Cobb-505 broiler strain). The chicks were obtained from a private farm at Kafrelsheikh Governorate, where five chicks were anesthetized (intraperitoneal injection of sodium pentobarbital (50 mg/kg) to minimize suffering during slaughtering) and slaughtered after weighting at the beginning of the experiment. Their bursa, spleen, and thymus gland were weighed. The remaining broiler chicks (120) were kept in a clean room, which was furnished with sawdust with good ventilation at the Animal Health Research Institute, Kafrelsheikh branch, where the experiment was done. Chicks were supplemented with feed and water ad libitum, and kept under good hygienic management and sanitation with light/dark 23/1 h./day. Chicks (average body weight = 40–50 g/chick), after 3 days acclimatization period were equally allotted into (ranking method), 4 groups each group contains 3 replicates (10 chicks/replicate), where the experiment was started at 4 days old. The negative control group (first group) was fed on a diet [40] without any additives (basal) or infection (Table 10). The positive control (second group) was fed on a diet without any additives but infected with *E. coli*. The third group was fed on agrimos**®** (2 kg/ton) supplemented diet but without infection. The fourth group was fed on agrimos**®** (2 kg/ton) supplemented diet and infected. All chicks groups were vaccinated against ND and IBD at 7 & 12 days, respectively. The chemical analysis of feed was determined according to AOAC [41]**.** Individual bird body weight was recorded at the starting of the experiment. All performance parameters (BW, weight gain (WG), and FCR) were calculated according to Vohra and Roudybush [42], Castell and Tiews [43]**,** and Tacon [44], respectively**.** The feed intake for each pen was recorded weekly with the observation of chicks for symptoms, PM, and mortality along the experimental period.

**Table 10:** Experimental feeding program

Physical composition	Basal diet (0–3) weeks	Basal diet (3–6) weeks
Yellow corn	55	61.35
Soybean meal 48%	32	30.6
Corn gluten 62%	4.42	0
Sunflower oil	4.4	4.4
Methionine	0.16	0.08
Dicalcium phosphate	1.85	1.4
Lime stone	1.25	1.3
Choline 60%	0.22	0.17
Common salt	0.4	0.4
Premix^a^	0.3	0.3
**Chemical composition %**		
ME Kcal/kg	3218.5	3229.7
Crude protein	23.02	20.03
Calcium	1	.9
Available phosphorus	0.45	0.35
Lysine	1.1	1
Methionine + cysteine	0.9	.72
Choline	1300 mg/1 kg	1000 mg/1 kg

^a^ The used premix (Multivita Co.) composed of retinol 12,000,000 IU, cholecalciferol 2,200,000 IU, tocopherol 10,000 mg, menadione 2000 mg, thiamin 1000 mg, riboflavin 5000 mg, pyridoxal 1500 mg, cobalamin 10 mg, Niacin 30,000 mg, Biotin 50 mg, Folic acid 1000 mg, Pantothenic acid 10,000 mg, Iron 30,000 mg, Manganese 60,000 mg, Copper 4000 mg, Zinc 50,000 mg, Iodine 1000 mg, Cobalt 100 mg, Selenium 100 mg, calcium carbonate (CaCO3) carrier to 3000 g

The sacrificed birds (Five birds from each group were anaesthetized by intraperitoneal injection of sodium pentobarbital (50 mg/kg) to minimize suffering during slaughtering) from the experimental groups were used for sample collection. The remaining live birds were suffocated in strong bags by CO2 suffocation. All birds (125), dead birds and slaughtered ones, as well as remnants of samples and bedding material, were buried in the strict hygienically controlled properly constructed burial pit.

### Experimental infection

At the starting of the experiment, all chicks in the two experimentally infected groups were infected with *E. coli* O_78_ via the oral route (1 ml containing 3 × 10^8^). This strain was supplied from the Animal Health Research Institute, Kafrelsheikh Regional Lab, Poultry, and Rabbit Disease Department). *E. coli* O_78_ was isolated from poultry and contained the virulence genes, which are a defining characteristic of Avian Pathogenic *E. coli* O_78_ (APEC). *E. coli* O_78_ was marked with an antibiotic (320 μg ciprofloxacin/ml broth) and prepared according to McFarland [45]. All groups (30 birds/group) were vaccinated against ND and IBD at days 7 and 12, respectively.

### Sample collection and measurements of the serum parameters

On day 35, blood (5 ml blood/bird) samples were collected from 5 birds randomly selected/group after slaughtering (the birds were anesthetized by intraperitoneal injection of sodium pentobarbital (50 mg/kg) to minimize suffering during slaughtering). The selection was not based on any pre-specified effect. After coagulation of the blood, serum was separated (centrifugation at 3000 rpm for 15 min.) and kept in a freezer (−20^o^ C) until analysis. The investigators were not blinded during data collection. Blinding was used during analysis. Computational analysis was not performed blinded. Serum activities of CAT, SOD, ALT, and AST and concentration of MDA, total protein, and albumin were measured by using commercial kits obtained from BIODIAGNOSTIC Company.

### Immune response against Newcastle disease (ND)

HI, titer was performed against ND [46]**.** The Newcastle disease virus and positive control serum were kindly provided by the Animal Health Research Institute, Kafrelsheikh.

### Colonization of *E. coli*

Shedding of the *E. coil* in the fecal matter and its colonization in organs was recorded and confirmed by the re-isolation of *E. coli* from infected birds.

### The weight of immune organs

Five chicks on zero-day of age and five chicks from each group were weighted then slaughtered on 15 and 35 days of age. Each organ including thymus, spleen, and bursa of Fabricius was isolated and weighed. The weight of the targeted immune organs was calculated as described by Keil et al. [47] following the equation below:

Immune Organ Weight/Body Weight = Organ Weight (gm)/Body Weight (gm) × 100.

### Necropsy and histopathology

Sections of liver, intestine, bursa, thymus gland, and spleen were taken after necropsy and fixed immediately in 10% buffered formalin and processed for histopathological evaluation, using routine paraffin embedding section. Sections of 3 μm thickness were cut and stained using H&E according to Bancroft and Gamble [48]**.**

### Q-PCR (real-time polymerase chain reaction)

#### Collection of the sample

On day 35, samples of the liver were collected on liquid nitrogen from 4 birds randomly selected/group into clean Eppendorf tubes and stored at − 80 °C until use.

### Q-PCR (real-time polymerase chain reaction)

The liver samples were collected (35 days) on liquid nitrogen from 4 birds/group into clean Eppendorf tubes and stored at − 80 °C until use.

Total RNA from the liver samples was extracted using easy RED total RNA extraction kits (iNtRON Biotechnology, Inc.) according to the manufacturer’s instructions. The RNA integrity was verified by using agarose gel electrophoresis. The first-strand cDNA was synthesized with total RNA using Intron-Power cDNA synthesis kit (Cat. no. 25011).

The specific primers were used to amplify the selected genes of the chicken (*Gallus gallus*) with GAPDH as housekeeping (internal standard) gene-primer sequence (Table 11). The qRT-PCR assay was carried out using a Stratagene MX300P Q-PCR system (Agilent Technologies), using Real MODTM Green FAST qPCR master mix (S) following the manufacturer’s recommendations. MxPro QPCR Software was used for data collection.

**Table 11:** Primers used for qPCR analysis

Primer	Sequence	Reference
**TNF-α**	for: 5′-GAGCTGTGGGGAGAACAAAAGGA-3′ Rev.: 5′-TTGGCCCTTGAAGAGGACCTG-3′	**Tabatabaei et al.** [49]
**NF-ϰB**	for: 5′-CAAGGCAGCAAATAGACGAG-3′ Rev.: 5′-GTTGAGAGTTAGCAGTGAGGCA-3’	
**GAPDH**	For- 5’CCTCTCTGGCAAAGTCCAAG3′ Rev- 5’CAACATCAAATGGGCAGATG3’	

TNF-α: Tumor necrosis factor-alpha, NF-ϰB: Nuclear factor-kappa B, GAPDH: Glyceraldehyde-3 phosphate dehydrogenase

The relative gene expression levels were evaluated using the 2 − ΔΔct method [50]**.**

### Statistical analysis

The obtained results were statistically analyzed by one-way ANOVA using **SPSS** version 16.

## Data Availability

The datasets used and/or analyzed during the current study are available from corresponding author on reasonable request.

## Abbreviations

AGP: Antibiotic Growth Promoters
ALT: Alanine transaminase
APPs: Acute-phase proteins
AST: Aspartate aminotransferase
BW: Body weight
CAT: Catalase
DNA: Deoxyribonucleic acid
*E. coli*: *Escherichia coli*
FCR: Feed conversion ratio
HI: Hemagglutination Inhibition
IBDV: Infectious bursal disease
Q-PCR: Real-Time Polymerase Chain Reaction
MDA: Lipid peroxidation
MOS: Mannan oligosaccharide
ND: Newcastle Disease
NDV: Newcastle disease virus
NF: Nuclear factor
PM: Postmortem
RNA: Ribonucleic acid
SOD: Superoxide dismutase
TNF: Tumor necrosis factor
